# Harmonization of whole-genome sequencing for outbreak surveillance of *Enterobacteriaceae* and *Enterococci*

**DOI:** 10.1099/mgen.0.000567

**Published:** 2021-07-19

**Authors:** Casper Jamin, Sien De Koster, Stefanie van Koeveringe, Dieter De Coninck, Klaas Mensaert, Katrien De Bruyne, Natascha Perales Selva, Christine Lammens, Herman Goossens, Christian Hoebe, Paul Savelkoul, Lieke van Alphen

**Affiliations:** ^1^​ Department of Medical Microbiology, Care and Public Health Research Institute (CAPHRI), Maastricht University Medical Center+, Maastricht, The Netherlands; ^2^​ Laboratory of Medical Microbiology, Vaccine & Infectious Disease Institute, University of Antwerp, Belgium; ^3^​ Laboratory of Clinical Microbiology, Antwerp University Hospital, Antwerp, Belgium; ^4^​ bioMérieux SA, Sint-Martens-Latem, Belgium; ^5^​ Department of Sexual Health, Infectious Diseases and Environment, South Limburg Public Health Service, Heerlen, The Netherlands

**Keywords:** bacterial typing, harmonisation, whole genome sequencing, ring-trial, nosocomial pathogens, antimicrobial resistance

## Abstract

Whole-genome sequencing (WGS) is becoming the de facto standard for bacterial typing and outbreak surveillance of resistant bacterial pathogens. However, interoperability for WGS of bacterial outbreaks is poorly understood. We hypothesized that harmonization of WGS for outbreak surveillance is achievable through the use of identical protocols for both data generation and data analysis. A set of 30 bacterial isolates, comprising of various species belonging to the *Enterobacteriaceae* family and *Enterococcus* genera, were selected and sequenced using the same protocol on the Illumina MiSeq platform in each individual centre. All generated sequencing data were analysed by one centre using BioNumerics (6.7.3) for (i) genotyping origin of replications and antimicrobial resistance genes, (ii) core-genome multi-locus sequence typing (cgMLST) for *Escherichia coli* and *Klebsiella pneumoniae* and whole-genome multi-locus sequencing typing (wgMLST) for all species. Additionally, a split *k*-mer analysis was performed to determine the number of SNPs between samples. A precision of 99.0% and an accuracy of 99.2% was achieved for genotyping. Based on cgMLST, a discrepant allele was called only in 2/27 and 3/15 comparisons between two genomes, for *E. coli* and *K. pneumoniae,* respectively. Based on wgMLST, the number of discrepant alleles ranged from 0 to 7 (average 1.6). For SNPs, this ranged from 0 to 11 SNPs (average 3.4). Furthermore, we demonstrate that using different *de novo* assemblers to analyse the same dataset introduces up to 150 SNPs, which surpasses most thresholds for bacterial outbreaks. This shows the importance of harmonization of data-processing surveillance of bacterial outbreaks. In summary, multi-centre WGS for bacterial surveillance is achievable, but only if protocols are harmonized.

## Data Summary

The authors confirm that all supporting data, code and protocols have been provided within the article. All raw sequencing data were deposited at EBI-ENA under BioProject PRJEB40571.

**Impact Statement**

Whole-genome sequencing (WGS) for typing bacterial outbreaks has surged in recent years. We performed an inter-laboratory ring-trial by sending out 30 bacterial isolates to assess the reproducibility of WGS. We demonstrated that the use of different *de novo* assemblers for a single outbreak analysis will lead to bacterial isolates being misclassified as not related to the outbreak cluster. Additionally, we show that implementing WGS for regional or (inter)national surveillance of bacterial pathogens is feasible if identical laboratory procedures and data analysis workflows are used.

## Introduction

The dissemination of antimicrobial resistance (AMR) has grown to an issue of worldwide proportions. Routine surveillance by molecular typing can aid in the fight against AMR, as outlined by the global action plan of the World Health Organization [1]. ESKAPE pathogens (*Enterococcus faecium, Staphylococcus aureus, Klebsiella pneumoniae, Acinetobacter baumannii, Pseudomonas aeruginosa* and *Enterobacter* species) are of major interest as they are the leading cause of hospital-related infections and outbreaks. Furthermore, reports show that the number of infections by resistant micro-organisms have been on the rise in recent years. Infections by multi-drug-resistant (MDR) bacteria are associated with an increase in economic burden [2] and negative patient outcomes such as morbidity and mortality [3, 4].

To determine the spread of resistance and of resistant microbes, different molecular typing methods are being applied. Older, established typing methods for outbreak surveillance, such as pulsed field gel electrophoresis (PFGE), amplification fragment length polymorphism (AFLP), multi-locus sequencing typing (MLST) and multi-locus variable-number tandem repeat analysis (MLVA) are slowly being replaced by whole-genome sequencing (WGS). The introduction of WGS to the field of bacterial typing and spread of AMR has set a new standard for discriminatory power and accuracy, as it encompasses a comprehensive view of the bacterial core and accessory genome. This gives rise to the possibility to determine clonal relatedness in a more discriminatory fashion, and at the same time provide data on resistance genes, plasmids and virulence-potential, which would otherwise require a combination of other methods [5–8]. Current methods to determine phylogeny are based on core-/whole-genome multi-locus sequence typing (cgMLST, wgMLST) [9, 10] or SNPs [11–13].

Approaches like cgMLST and wgMLST determine the phylogeny among bacterial isolates based on differences in allelic profile in either the core genome or the entire genome, respectively. All coding sequences (CDS) or loci are identified using tools such as Prodigal [14]. Then, all variants of each locus are assigned a unique allele number and the complete set of allele numbers is called the allelic profile. The genetic distance is calculated by counting the number of discrepant alleles between two isolates. A relative genetic distance can also be calculated by dividing the number of discrepant alleles by the number of alleles that were compared. Next to commercial packages for cgMLST and wgMLST analyses, such as BioNumerics or SeqSphere, open source options are available as well, such as ChewBBACA [10] and Enterobase [15].

Inferring phylogeny based on SNPs can be performed by three different methods. (i) Alignment to a reference genome (Snippy [11]). (ii) (Core-) genome alignment (MAUVE [16] or Harvest Suite [17]). (iii) Alignment-free methods based on using the entire collection of subsequences of a sequence of length *k*: *k*-mer (kSNP [18] or SKA [13]).

Currently, only a few studies have described clonal-cluster thresholds definitions using cgMLST, wgMLST or SNP-based methods. Generally, these studies determine the thresholds based on either (i) previous or ongoing bacterial outbreaks in hospitals and in the food production chain, or (ii) by means of follow-up on human carriers of these pathogens over time. Furthermore, most of these studies only describe single-clone outbreaks, which can hamper the interpretation when these thresholds are applied to different lineages of a specific species. Some clinically relevant lineages might be more clonal than others, and so require different thresholds. One of the first reports on the use of WGS for bacterial outbreak analysis were on methicillin-resistant *Staphylococcus aureus* (MRSA) in 2013, in a neonatal intensive care unit. Next to standard assessment of epidemiological data and antibiograms, WGS was performed to resolve this putative outbreak [19]. In that study, a maximum of 20 SNPs was observed among the MRSA isolates found in the outbreak. For the foodborne pathogen *E. coli* O157:H7, the Public Health Agency Canada evaluated WGS for outbreak detection [20]. To this end, they retrospectively performed WGS for 250 isolates, from eight different outbreaks and analysed using wgMLST and SNP analyses. These 250 isolates were previously typed using MLVA or PFGE. WGS-based typing was in excellent concordance with MLVA and PFGE and also had higher discriminatory power to resolve outbreak clusters. Additionally, they reported that all isolates for each outbreak fell within a cutoff of 5 SNPs or 10 allele differences (on wgMLST basis). In their review, Schürch *et al.* suggested various clonal-cluster thresholds based on wgMLST or SNP analyses for a few common bacterial pathogens in outbreak situations [9].

Kluytmans-Van Den Bergh *et al.* recently determined clonal-cutoffs based on cgMLST and wgMLST for four extended-spectrum beta-lactamase-producing *Enterobacteriaceae* (ESBL-E)*: E. coli, K. pneumoniae*, *Citrobacter* species and *Enterobacter* sp*.* [21]. In their study, isolates were classified as epidemiologically linked when these were cultured from a single patient in a 30 day time window and when they belonged to the same seven-gene sequence type. Subsequently, the genetic distance (here defined as number of discrepant alleles divided by the number of alleles compared) was compared among all isolates, and clonal thresholds were determined by the lowest genetic distance possible that included all epidemiologically linked isolates.

The goal of the i-4-1-Health study is to assess the prevalence and spread of resistant bacteria among humans and animals in the Dutch-Belgian border [22]. Across a 1 year period, we screened patients in hospitals and in long-term healthcare facilities, infants at day-care facilities, and broilers and weaned pigs for gut or rectal carriage of ESBL-producing, ciprofloxacin-resistant or carbapenemase-producing Enterobacteriaceae and vancomycin-resistant *Enterococci*. This One-Health approach could provide insights into the prevalence and spread of resistant bacteria between and within these separate domains. In the i-4–1-Health study, WGS data was generated in three independent locations, and thus inter-laboratory reproducibility needed to be assessed to allow the comparison of this data. To standardize the WGS results and interpretation, we made efforts to harmonize the WGS protocols, both for the wet-lab procedures and the bioinformatics analysis.

Here, we harmonized the inter-laboratory reproducibility of WGS for outbreak surveillance and genotyping of AMR and origin of replication (ORI) of plasmids for a selection of AMR bacteria frequently encountered in hospital-related infections and AMR surveillance within the i-4-1-Health project. As the implementation of WGS for routine outbreak surveillance is particularly dependent on standardized methodology, we evaluated the technical variation in phylogenetic comparison using a commercially available wgMLST tool in BioNumerics and an open-source reference-free SNP-based tool called SKA [13].

## Methods

### Selection of isolates

In total 30 resistant bacterial isolates were selected based on their extended-spectrum beta-lactamase (ESBL) or carbapenemase activity, or based on ciprofloxacin or vancomycin resistance phenotype. The complete collection of isolates consisted of nine *E. coli*; five *K. pneumonia*; four *Citrobacter* sp*.*; four *Enterobacter* sp*.;* two *Klebsiella oxytoca;* two *Klebsiella aerogenes;* two *Enterococcus faecalis* and two *E. faecium*. Six isolates (two *E. coli,* two *K. pneumoniae* and two *Enterobacter* sp*.*) were collected previously [21] and kindly provided by the SoM study-group, and 20 isolates were collected during the i-4-1-Health study [22]. The *E. faecium* and *E. faecalis* isolates were from a previous collection, stored at Antwerp University. The isolates were collected from perianal swabs of hospitalized patients (21) and clients in nursing homes (6), and from faeces from broilers (2) and weaned pigs (1) by selective culturing. The culturing methods are described elsewhere [21, 22]. An overview of isolates and their origin is available in Table 1. Isolates were inoculated from −80 °C on Mueller–Hinton II agar (BD, Franklin Lakes, NJ, USA) and sent to the participating institutes. The 30 isolates were divided in three sets of ten isolates. Each set was sequenced once by each centre, with a 6 month interval between each set.

**Table 1.** Metadata of all isolates used in this study

Name	Species	Origin	Study	County	Accession no. centre 1	Accession no. centre 2	Accession no. centre 3
***Citrobacter*** **sp. 1**	*Citrobacter* sp.	Hospital	i-4-1-Health	Netherlands	ERS5219870	ERS5219871	ERS5219872
***Citrobacter*** **sp. 2**	*Citrobacter* sp.	Long-term healthcare facility	i-4-1-Health	Netherlands	ERS5219873	ERS5219874	ERS5219875
***Citrobacter* sp. 3**	*Citrobacter* sp.	Long-term healthcare facility	i-4-1-Health	Netherlands	ERS5219876	ERS5219877	ERS5219878
***Citrobacter*** **sp. 4**	*Citrobacter* sp.	Hospital	i-4-1-Health	Netherlands	ERS5219879	ERS5219880	ERS5219881
***Enterobacter*** **sp. 1**	*Enterobacter* sp.	Long-term healthcare facility	i-4-1-Health	Netherlands	ERS5219882	ERS5219883	ERS5219884
***Enterobacter*** **sp. 2**	*Enterobacter* sp.	Hospital	i-4-1-Health	Netherlands	ERS5219885	ERS5219886	ERS5219887
***Enterobacter*** **sp. 3**	*Enterobacter* sp.	Hospital	SoM	Netherlands	ERS5219888	ERS5219889	ERS5219890
***Enterobacter*** **sp. 4**	*Enterobacter* sp.	Hospital	SoM	Netherlands	ERS5219891	ERS5219892	ERS5219893
***E. coli 1***	* E. coli *	Hospital	SoM	Netherlands	ERS5219828	ERS5219829	ERS5219830
***E. coli 2***	* E. coli *	Hospital	SoM	Netherlands	ERS5219831	ERS5219832	ERS5219833
***E. coli 3***	* E. coli *	Hospital	i-4-1-health	Netherlands	ERS5219834	ERS5219835	ERS5219836
***E. coli 4***	* E. coli *	Hospital	i-4-1-Health	Netherlands	ERS5219837	ERS5219838	ERS5219839
***E. coli 5***	* E. coli *	Broiler	i-4-1-Health	Netherlands	ERS5219840	ERS5219841	ERS5219842
***E. coli 6***	* E. coli *	Weaned pig	i-4-1-Health	Netherlands	ERS5219843	ERS5219844	ERS5219845
***E. coli 7***	* E. coli *	Long-term healthcare facility	i-4-1-Health	Netherlands	ERS5219846	ERS5219847	ERS5219848
***E. coli 8***	* E. coli *	Broiler	i-4-1-Health	Netherlands	ERS5219849	ERS5219850	ERS5219851
***E. coli 9***	* E. coli *	Hospital	i-4-1-Health	Netherlands	ERS5219852	ERS5219853	ERS5219854
***E. faecalis 1***	* E. faecalis *	Hospital		Belgium	ERS5219894	ERS5219895	ERS5219896
***E. faecalis 2***	* E. faecalis *	Hospital		Belgium	ERS5219897	ERS5219898	ERS5219899
***E. faecium 1***	* E. faecium *	Hospital		Belgium	ERS5219900	ERS5219901	ERS5219902
***E. faecium 2***	* E. faecium *	Hospital		Belgium	ERS5219903	ERS5219904	ERS5219905
***K. aerogenes 1***	* E. aerogenes *	Hospital	i-4-1-Health	Belgium	ERS5219912	ERS5219913	ERS5219914
***K. aerogenes 2***	* E. aerogenes *	Hospital	i-4-1-Health	Belgium	ERS5219915	ERS5219916	ERS5219917
***K. oxytoca 1***	* K. oxytoca *	Hospital	i-4-1-Health	Belgium	ERS5219906	ERS5219907	ERS5219908
***K. oxytoca 2***	* K. oxytoca *	Hospital	i-4-1-Health	Netherlands	ERS5219909	ERS5219910	ERS5219911
***K. pneumoniae 1***	* K. pneumoniae *	Hospital	i-4-1-Health	Belgium	ERS5219855	ERS5219856	ERS5219857
***K. pneumoniae 2***	* K. pneumoniae *	Long-term healthcare facility	i-4-1-Health	Netherlands	ERS5219858	ERS5219859	ERS5219860
***K. pneumoniae 3***	* K. pneumoniae *	Long-term healthcare facility	i-4-1-Health	Netherlands	ERS5219861	ERS5219862	ERS5219863
***K. pneumoniae 4***	* K. pneumoniae *	Hospital	SoM	Netherlands	ERS5219864	ERS5219865	ERS5219866
***K. pneumoniae 5***	* K. pneumoniae *	Hospital	SoM	Netherlands	ERS5219867	ERS5219868	ERS5219869

### DNA isolation and WGS

The DNA isolation and WGS procedure was performed as follows: DNA was extracted using the MasterPure DNA isolation kit (Lucigen) or MasterPure Gram Positive DNA purification kit (Lucigen). Sequencing libraries were prepared using NexteraXT (Illumina). Libraries were sequenced on the Illumina MiSeq platform in paired end 2×250 base-pair (bp) reads using the MiSeq V2 cartridge. Where possible, each set of isolates was subjected to WGS in a single run. Acceptance criteria for WGS were a *de novo* assembly with an average coverage higher than 30 and less than 1000 contigs, as reported in BioNumerics (7.6.3). Samples not fulfilling acceptance criteria were re-sequenced. The accession numbers for the raw sequencing data are available in Table 1. Analysis of the generated datasets (*n*=90) was performed in one institute.

### cgMLST and wgMLST allele calling and genotyping

Raw sequencing reads were assembled using a custom pipeline in BioNumerics (7.6.3) employing SPAdes [23] (v3.7.0) for itsde *novo* assembly. From the raw reads and the *de novo* assembly, alleles were called for core-genome and whole-genome MLST (cgMLST/wgMLST). In BioNumerics, cgMLST schemes were only available for *E. coli* and *K. pneumoniae* consisting of 2513 and 634 fixed loci, respectively. Pairwise allelic distance was determined by counting the number of discrepant allele variants between two datasets, ignoring loci that were not present in both datasets. Resistance genes and origins of replication (ORI) were determined using blast [ 24] and two custom databases based on Resfinder [25] and PlasmidFinder [26]. AMR genes were called with a using 90% identity and 60% length cutoff. ORIs were called using 95% identity and 60% length cutoff. In total, 90 WGS datasets were generated. As no gold standard with regard to true genotype of each isolate was available, the following rules were applied: (i) if either two or three out of three datasets of an isolate had a specific genotype, this was considered as a true positive observation; (ii) if only one out of three datasets of an isolate had a specific genotype, this was considered as a false positive observation; (iii) if a different allelic variant was observed (i.e.two blaTEM-1B and one blaTEM-116) this was noted as a discrepancy and counted as a false positive.

### wgSNP analysis

To determine the best *de novo* assembler to use for wgSNP analysis, we chose the assembler generating the least amount of pairwise SNPs (using SKA), among assemblies of the same isolate. To avoid complexity, only the *E. coli* dataset of this study was used. The following assemblers were used: (I) SPAdes (v3.14.0) [23], (II) SKESA (parameters:‘—use-paired_end’, v2.3.0) [27], (III) Megahit [28] (v1.2.9). All tools were used in default settings, unless otherwise specified. Additionally, the assembly-free method to determine SNPs straight from the raw reads, using ‘SKA fastq’, was also used in this comparison. The complete workflow is available at ‘https://github.com/MUMC-MEDMIC/assemblercompare’ (v1.0). SKA [13] was used to determine SNPs on a whole-genome level, using a splitk-mer length of 31. In short, pairwise SNPs were determined by generating a profile of split k-mers, in which the middle base may vary (‘SKA fasta’ for assembly- or ‘SKA fastq’ for read based SNP profiling). The number of SNPs, between two datasets, was determined by comparing the split k-mer files (‘SKA distance’). All data preprocessing for the SNP-based data analysis was performed using Snakemake [29] as the workflow manager.

### Statistical analysis

Statistical analyses were done using scipy.stats module (V1.3.1) [30] and the statsmodel.api package in Python (v3.7).

## Results

**Fig. 1.**
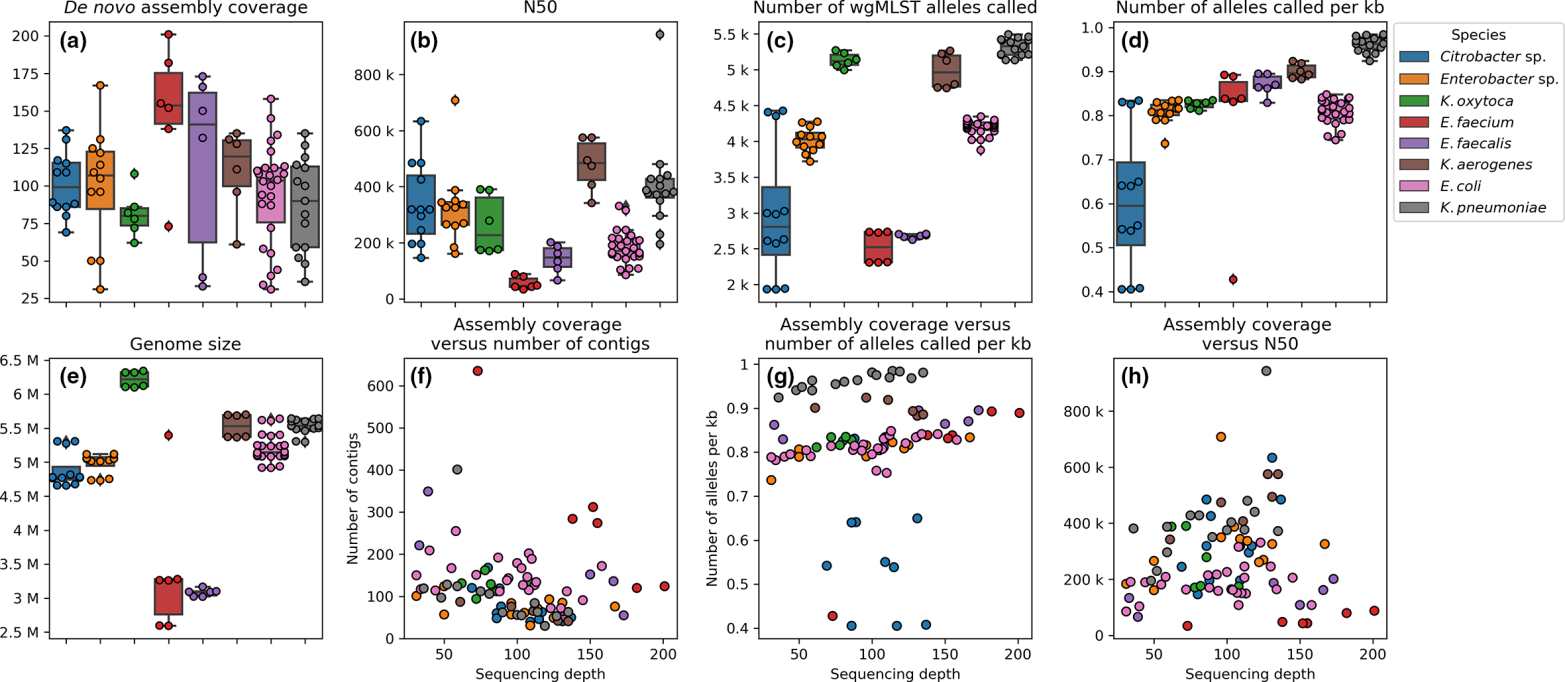
Distribution of various quality parameters pre and post *de novo* assembly. Subplots (a–e) show boxplots with interquartile (IQ) range. Whiskers range up to 1.5 times the IQ range. All single datapoints are represented as single dots. Subplots (f-h) show scatterplots of relations between two quality metrics.

The assembly coverage, or the depth of coverage, of all isolates ranged from 30 to 203 (Fig. 1a). The N50 score, indicative for how fragmented a *de novo* assembly is, ranged from 33712 bp (*E. faecium*) to 942715 bp (*K. pneumoniae*) and showed clear species dependence (Fig. 1b). Assemblies of *E. coli, E. faecalis* and *E. faecium* showed a lower N50 score, indicating the difficulties of assembling such genomes (Fig. 1b). The number of contigs also varied per species, and overall had a significant negative correlation with the sequencing depth (*P*<0.01, Spearman rank correlation, Fig. 1f).

The number of wgMLST alleles called ranged from 1933 (*Citrobacter* sp*.)* alleles to 5493 (*K. pneumoniae*, Fig. 1c). Furthermore, the average number of alleles per kilobase (kb) ranged from 0.41 to 0.98. A significant positive correlation between the normalized allele count and sequencing depth was observed (*P*<0.05, Spearman rank correlation Fig. 1g). Surprisingly, the *Citrobacter* sp*.* datasets seemed to showed a low coding density (range 0.41 to 0.65) compared to the median of the entire dataset (0.83). Further inspection of the *Citrobacter* sp*.* genome assemblies using blast webservice (https://blast.ncbi.nlm.nih.gov/Blast.cgi, accessed 1 April 2020), showed low homology (~85% DNA identity score) to known *Citrobacter* sp*.* isolates available in the NCBI database (accessed on 1 Aril 2020, data not shown).

One dataset of *E. faecium-1* had an unusually large genome size of 5.4 Mb (Fig. 1e). This dataset also had a higher number of contigs; (636, median of 274 for *E. faecium,* Fig. 1f *)*, and showed a lower number of alleles per kb (0.43, median of 0.84. Fig. 1d) compared to the other *E. faecium* datasets. This indicates contamination in the NGS dataset of a non- *E. faecium* microbe. Manual inspection of the assembly, using blast webservice, showed the presence of contigs belonging to *Cutibacterium* (formerly known as *Propionibacterium*), a skin commensal and previously described as a common contaminant of NGS datasets [31–33].

### Resistance genes and plasmid ORIs

**Fig. 2.**
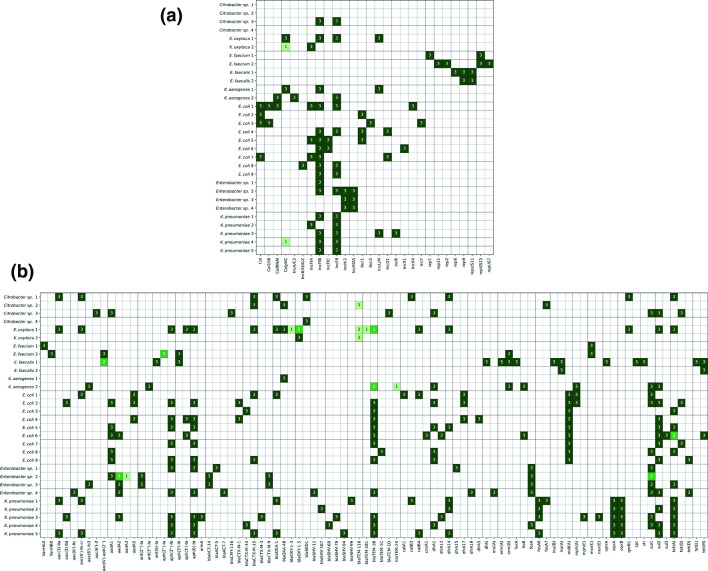
(a) Heatmap of the number of genotype calls for various origins of replication among the isolates subjected to WGS. Genotype calls per locus was summed up for each centre’s isolate if this locus was detected in their dataset. (b) Heatmap of the number of genotype calls for various AMR genes, among the isolates subjected to WGS. Genotype calls per locus was summed up for each centre’s isolate if this locus was detected in their dataset.

Overall, a good consensus was obtained for the genotyping of plasmid ORIs and AMR genes (Fig. 2a, b). A total of 973 AMR genes and ORIs were called with a precision of 99.0% and sensitivity of 99.2%. For four isolates, a genotype was not called in one of the datasets. The missed genotypes were for *E. cloacae-2* a *sul1* gene, for *E. coli-6* a *tet(A)* gene, for *E. faecalis-*1 an *aac(6')-aph(2')* gene, and for *E. faecium-2* an *aph(2'')-Ia* gene. For *Citrobacter* sp*.-2* and *K. oxytoca-2,* a false discovery of a *blaTEM-116* was observed, as this genotype was not called in either of the other two datasets of these isolates. For four isolates, a discrepant genotype was called. These discrepancies were observed for *K. aerogenes-2* (*blaTEM*), for *E. cloacae-2* (*aadA*), and for *K. oxytoca-1* (*blaOXY* and *blaTEM*). Twice, an unexpected ColpVC was found in a *K. oxytoca-2* and *K. pneumoniae-4* dataset, which were from two different centres, indicating either loss of this plasmid in the other dataset of this isolate, or contamination during DNA isolation or library preparation (Fig. 2a).

### Inter-laboratory variation in cgMLST profiles

To assess the baseline genetic variation of identical isolates when these isolates were sequenced in different sequencing institutes, we compared the cgMLST and wgMLST profiles among the isolates from the three participating institutes. Only for *E. coli* and *K. pneumoniae* cgMLST schemes were available for use in BioNumerics. On average, 2441 (97.1%) and 615 (97.1%) core-genome alleles were called for *E. coli* and *K. pneumoniae* respectively (Fig. S1, available in the online version of this article). In total, 27 and 15 pairwise allelic distances were calculated among the nine *E. coli* and five *K. pneumoniae* isolates. In 25/27 (93%) and 12/15 (80%) comparisons, a perfect concordance of cgMLST profiles was observed. If no concordance in cgMLST profiles was observed, only one allele was differently called (Fig. S2).

### Inter-laboratory variation in wgMLST profiles

**Fig. 3.**
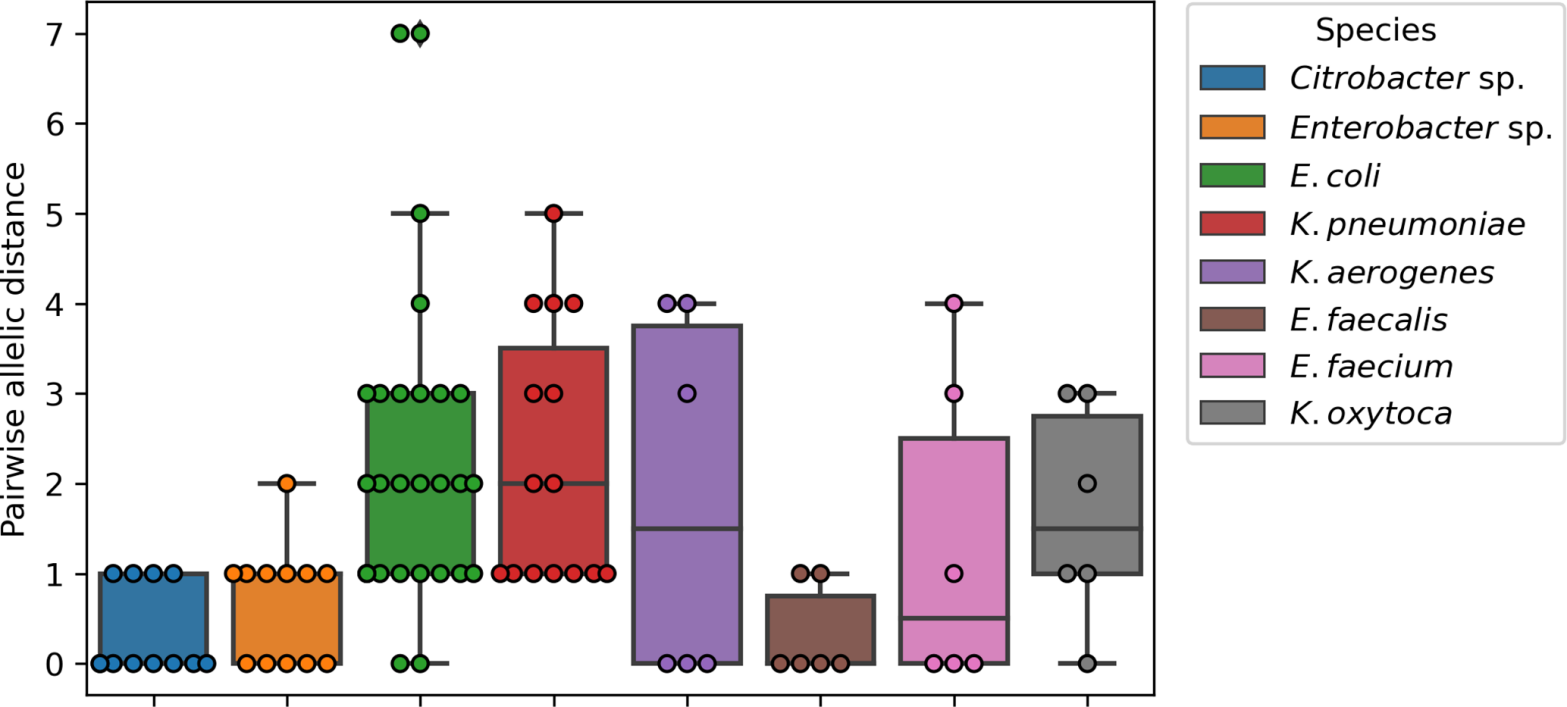
Boxplot of the allelic distance based on wgMLST between the triplicates that were selected for WGS. Boxes show IQ range and whiskers range up to 1.5 times the IQ range. All single pairwise observations were plotted as dots.

In total 90 pairwise comparisons were made for *K. oxytoca* (6), *Citrobacter sp.* (12), *E. coli* (27), *K. pneumoniae* (15), *E. cloacae* (12), *K. aerogenes* (6), *E. faecalis* (6) and *E. faecium* (6). Perfect concordance in wgMLST profiles was obtained in 26/90 (29%) comparisons (Fig. 3). In 44/90 (49%) pairwise comparisons, one or two discrepant alleles were observed. Only 23/90 (22%) comparisons showed more than two discrepant alleles, with a maximum of seven alleles different for an *E. coli*. For *E. faecium-1* with the contamination of *Cutibacterium* had a perfect concordance of wgMLST profiles was observed (data not shown). This indicates the robustness of allele-based typing despite contamination with bacterial DNA from different species. For all species, an average allelic distance of 1.6 alleles (standard deviation 1.6) was observed.

For the four *Citrobacter* sp., a highly diverse number of wgMLST alleles were called, ranging from 1933 to 4426. The genome size did not vary strongly (mean 4.88 Mb, range 4.66 Mb to 5.31 Mb). The normalized allele counts were lower for *Citrobacter* sp*.* (mean 0.61, range 0.41 to 0.83) than in other species in this study (mean 0.84, range 0.43 to 0.98). Therefore, the variation in the number of alleles in the wgMLST scheme for *Citrobacter* sp*.* cannot be determined in this study, as an incomplete set of alleles were called.

### Reference free wgSNP

As mutations in the genome can also arise in intergenic regions (which are not taken into account in MLST-based methods), all assemblies of each isolate were screened using pairwise SNPs. First, the most optimal assembler for this task was chosen. For this, we determined the inter- and intra-assembler variation introduced on the number of pairwise SNP between two *de novo* assemblies. The best assembler was chosen based on the one that introduced the least number of pairwise SNPs in the datasets from the same isolates with the intra-assembler comparison. To reduce complexity, only the *E. coli* dataset was used. Secondly, the number of pairwise SNPs was determined for the entire dataset using the best suited assembler. Additionally, we also used the assembly-free method for determining SNPs, as in implemented by SKA.

**Fig. 4.**
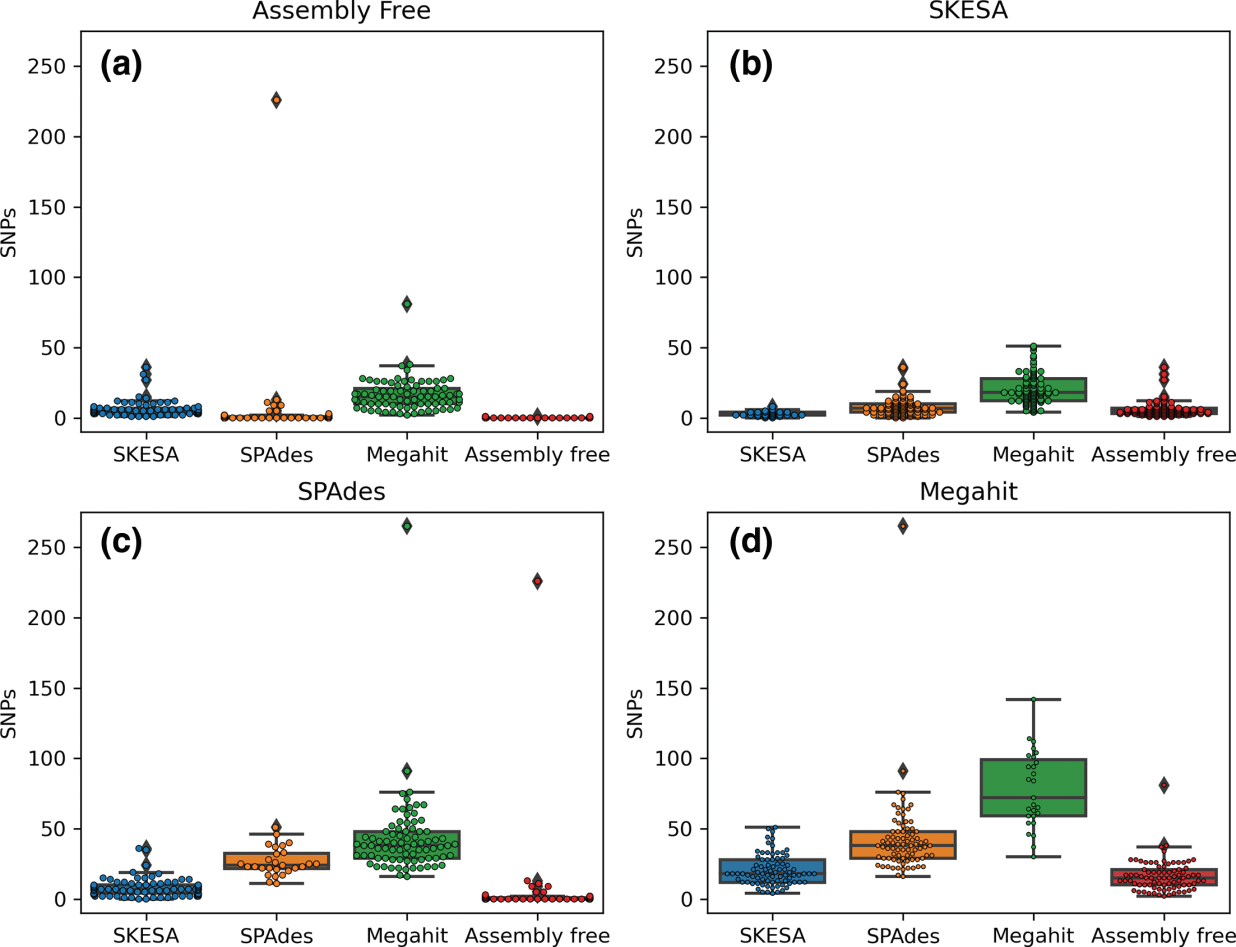
Boxplots of the inter- and intra-assembly difference in *de novo* assemblies based on SNPs, using SKA for the *E. coli* dataset. *De novo* assembly method compared to is indicated above each box. (a) Assembly free, (b) SKESA, (c) SPAdes and (d) Megahit. Boxes show IQ range. Whiskers range up to 1.5 times the IQ range. All single pairwise observations were plotted as dots.

The mean intra-assembly variation was 0.2 SNPs (assembly free), 2.7 SNPs (SKESA), 26.6 SNPs (SPAdes) and 77.8 SNPs (Megahit) (Fig. 4a–d). The mean inter-assembler variation ranged from 3.9 (assembly free compared to SPAdes) up to 43.0 SNPs (‘SPAdes to megahit’). All combinations, except the ‘assembly-free to assembly-free’ and ‘SKESA to SKESA’, revealed pairwise comparisons with over 20 SNPs for the *E. coli* dataset. Therefore, only these two methods were used to analyse the complete dataset.

**Fig. 5.**
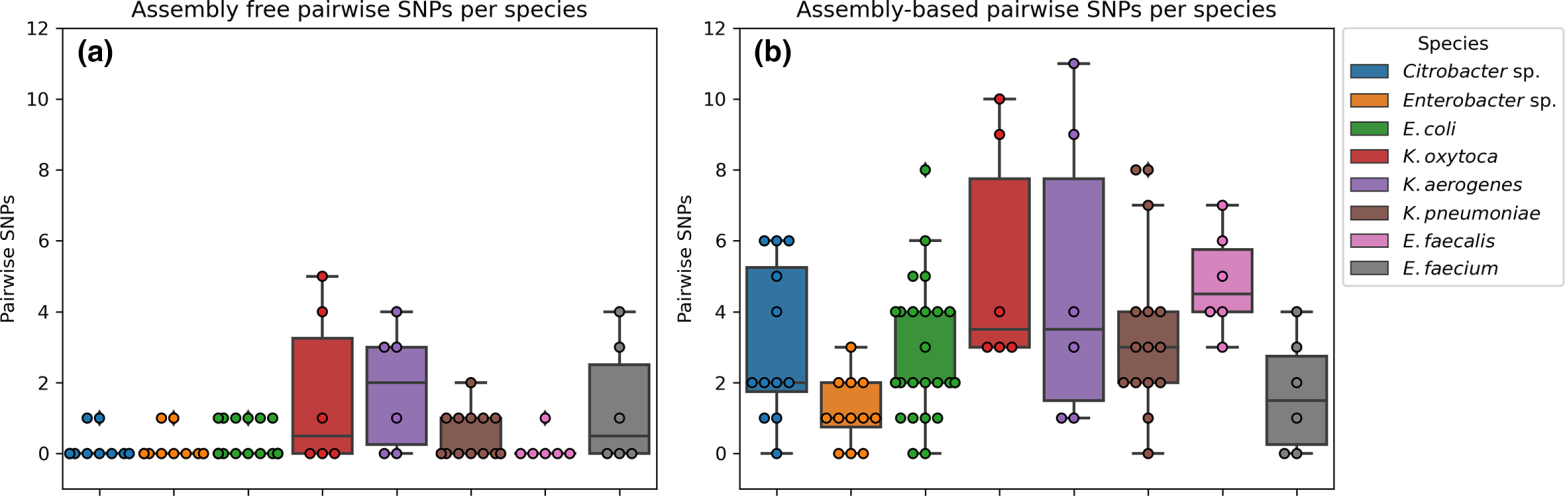
Boxplot of the SNP distance between the triplicates that were selected for WGS. Boxes show IQ range and whiskers range up to 1.5 times the IQ range. All single pairwise observations were plotted as dots. (a) SNP distances using the raw reads as input for SKA. (b) SNP distances based on the *de novo* assembly using SKESA.

Using the assembly-free approach, 63/90 (70%) and 21/90 (21%) comparisons show zero or one pairwise SNP, respectively (Fig. 5a). Only for *K. pneumoniae*, *E. faecium, K. oxytoca* and *K. aerogenes* was more than one pairwise SNP observed, with a maximum of five SNPs for *K. oxytoca*. Using the assembly-based approach zero SNPs were observed among assemblies in 10/90 (10%) comparisons (Fig. 5b). Less than five pairwise SNPs were observed in 72/90 (80%) of the comparisons. Interestingly, in the *K. aerogenes* and *K. oxytoca* datasets, more than eight pairwise SNPs were observed. However, on wgMLST no more than four alleles' difference was observed. On average, 3.4 (standard deviation 2.6) pairwise SNPs were observed between assemblies of the same isolates (but sequenced in different institutes). Overall, more pairwise SNPs were observed when assemblies were used for SNP analysis compared to screening raw reads for SNPs. The difference in number of *k*-mers between the assemblyfree and assembly-based methods ranged from −2.1–1.2% (Fig. S3), indicating that a similar amount of *k*-mers were compared in both methods.

## Discussion

Using an inter-laboratory ring trial we evaluated the reproducibility of whole-genome sequencing for outbreak surveillance purposes. Participating institutes subjected the same set of 30 bacterial isolates of various *Enterobacteriaceae* and *Enterococci* species to whole-genome sequencing. As a first step, we assessed various QC measures and observed a slight positive trend of the sequencing depth on the normalized number of alleles called in the sequencing depth range of 30 to 207-fold. It remains unclear what sequencing depth is needed to correctly reconstruct the maximum possible number of correct alleles in the genome. Kluytmans-Van Den Bergh *et al.* [21] demonstrated an increase in resolution for phylogenetic reconstruction of *Enterobacteriaceae* if wgMLST is implemented compared to cgMLST. This would indicate that making more alleles available for comparison will improve the surveillance of outbreaks by cgMLST or wgMLST methods. Therefore, it is advisable to generate WGS data of sufficient depth to maximize the number of loci in the *de novo* assembly. On the other hand, deeper sequencing after a certain depth may not improve the phylogenetic signal any further, and does increase the run-time of subsequent *de novo* assembly.

Prokaryotes show a coding density of 1 CDS per 1 kb [34], however we observed a lower allele density. The majority of our datasets showed a lower number allele density (0.83 per kb, Fig. 1d), which could be caused by the quality-filtering step in allele calling. However, the low number of called alleles for most *Citrobacter* sp*.* may be explained by incomplete allele schemes, which do not contain the complete diversity of alleles. This indicates that the diversity of *Citrobacter* sp*.* genome assemblies present in public databases is incomplete, and our data may represent the discovery of a new antibiotic-resistant *Citrobacter* sp. in The Netherlands.

### Genotyping AMR genes and ORIs

We next performed identification of AMR genes and plasmid ORIs. Overall, an excellent reproducibility was achieved, as a precision of 99% was obtained. Most discrepancies could be explained by the variation in the variant calling of a specific resistance gene. There was an unexplained absence of a resistance gene four times in 973 genotype calls. Although the DNA isolation method used here showed good results for the application of WGS [35], some loci could still be missed due to inefficient isolation of plasmid DNA, where these AMR genes can be located. Only twice, and in different institutes, an unexpected ColpVC ORI was found in one of the sequencing datasets, which may indicate contamination during DNA isolation or library preparation. Strauß and co-authors reported a 1.7% discordance between WGS and micro-array for the detection of resistance and virulence genes [36]. In this study, a similar reproducibility in typing resistance genes and ORIs was obtained and previously described by Kozyreva *et al.,* which found a reproducibility rate of 99.97% [37].

### Genetic variation

It is of great importance that the genetic distance between technical duplicates does not surpass commonly used thresholds to classify isolates into clusters. In this study some variation among the wgMLST allelic profiles was observed, translating to an average of 0.49 discrepant allele per 1000 alleles. Kluytmans-Van Den Bergh *et al.* [21] reported a variation in genetic distance based on wgMLST in a range of 0 to 0.001 (which translates to five alleles difference, based on 5000 alleles compared) for five *E. coli* and three *K. pneumoniae,* which were sequenced in duplicate [21]. This is in concordance with our study, where 88/90 comparisons differed by no more than five alleles. Additionally, clonal thresholds reported by these authors were roughly 26 and 2 alleles difference for *E. coli* and *K. pneumoniae* on cgMLST, respectively. For wgMLST this was 29, 23, 8 and 14 alleles difference for *E. coli, K. pneumoniae*, *Citrobacter* sp*.* and *Enterobacter* sp*.,* respectively. These clonal thresholds are higher by a safe margin than the variation between any of the replicates in our study presented here. Although variation on a genetic level was observed, the level of disparity remained below other thresholds commonly employed for hospital outbreak surveillance purposes [9]. Previous work suggested a cut-off of 10 alleles for MDR *E. coli* and *K. pneumoniae* based on cgMLST [38, 39]. Therefore, it is safe to assume that if harmonized protocols are used, the technical genetic variation will remain within these previously described thresholds.

In the wgSNP analysis, all methods except for the ‘assembly-free to assembly-free’ and ‘SKESA to SKESA’ showed pairwise comparisons with more than 20 SNPs. This indicates that using SPAdes or Megahit in combination with a SNP-based method is unsuitable for outbreak surveillance, as datasets from identical isolates have more SNPs than commonly used outbreak thresholds [9], indicating that these isolates would be considered not clonally related, thus not belonging to the same outbreak. Furthermore, this also held true when comparing two assembly methods, which implies that comparing bacterial assemblies should be avoided at all costs if centres employ different methodologies to generate *de novo* assemblies for WGS outbreak surveillance. Potential outbreaks could be missed due to the large number of SNPs detected, resulting in identical isolates not being flagged as clonally related. This would subsequently have implications for infection prevention and control. For the assembly-free method, we observed most replicates to have no SNPs between each other (70%), which is in line with the GenomeTrakr proficiency-test study, which found a similar fraction of datasets showed having no SNPs (73%) [40].

Variation in SNPs among isolates showed a lower number of SNPs based on the assembly-free method compared to the assembly-based method. It is unlikely that this is caused by different numbers of *k*-mers that were compared for SNPs, as there was only a modest difference for the number of *k*-mers compared between the assembly-free and the assembly-based SNP analysis, which ranged from −2.1–1.2% difference in compared *k*-mers (Fig. S3). Therefore, it is more likely that *de novo* assembly introduces phylogenetic noise in regions difficult to assemble, like regions such as mobile elements (transposons and plasmids). Previously described work employing SNP-based methodologies to infer phylogeny among bacterial isolates often mask regions in the genome that are sensitive for non-informative SNPs for phylogenetic reconstruction, such as mobile genetic elements (MGE). Masking of these regions requires specialized tools such as Gubbins [41] that are able to recognize regions with elevated numbers of base substitutions in the genome. Unfortunately, using this reference-free methodology makes this masking impossible to perform in an unbiased and automated fashion like in the Gubbins pipeline. Therefore, we must assume the possibility of overestimation of SNPs among isolates in our study.

### Study limitations

For this study, only three centres participated in this ring-trial, all part of the i-4-1-Health study group. Here, ESBL-producing and ciprofloxacin-resistant *Enterobacteriaceae* and vancomycin-resistant *Enterococcus* were defined of primary interest, however other important nosocomial bacterial pathogens such as *Pseudomonas* sp., *Staphylococci* and A*cinetobacter* sp*.* were not included in the study. Furthermore, all three centres used the same protocols for the extraction and library preparation for sequencing on the Illumina MiSeq. Recommendations for future research would therefore be to determine if these harmonized wet-lab protocols and subsequent bioinformatic data processing are indeed required for the reconstruction of outbreak clusters.

### Conclusion

Overall, the work presented here demonstrated that whole-genome sequencing generates reproducible results when comparing results across laboratories that use identical wet-lab and dry-lab methodologies for WGS. Furthermore, to make multi-centre outbreak surveillance feasible in the future, we recommend that laboratories share raw sequencing reads, because systematic errors were introduced in the *de novo* assemblies by different assemblers. Finally, work presented here lays the foundation for routine proficiency testing in clinical microbiology laboratories.

## Supplementary Data

Supplementary material 1
