# Supplementary material for: Harmonization of whole-genome sequencing for outbreak surveillance of Enterobacteriaceae and Enterococci
Source: Microb Genom. 2021 Jul 19;7(7):000567. doi: 10.1099/mgen.0.000567 (PMC8477410; doi:10.1099/mgen.0.000567)
Supplement: Supplementary material 1 [file mgen-7-0567-s001.pdf]

Supplemental figures

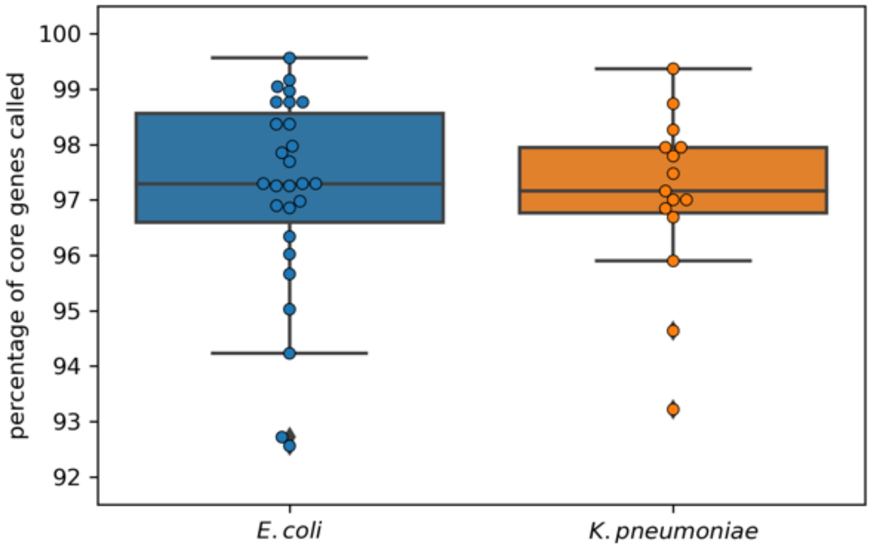

Supplemental figure 1. Boxplot of the number of core genes called for cgMLST in percentage for *K. pneumoniae* and *E. coli* respectively. Boxes range the interquartile (IQ) range. Whiskers range up to 1.5 times the IQ range. All single datapoints are represented as single dots. Only cgMLST schemes were available for *E. coli* and *K. pneumoniae.*

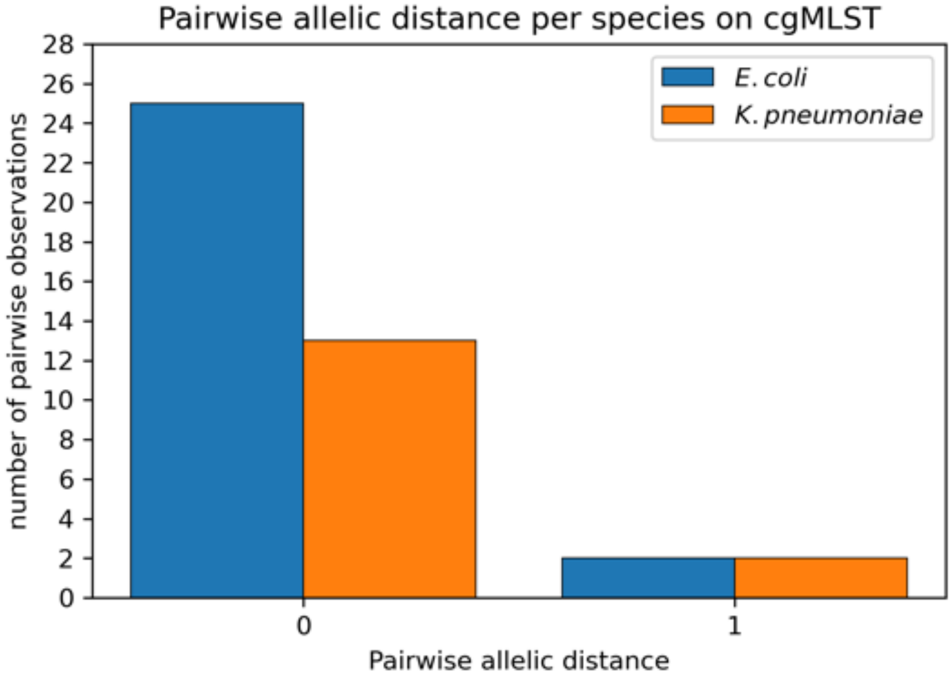

Supplemental figure 2. Barplot of the pairwise number of alleles that were different between two strains.

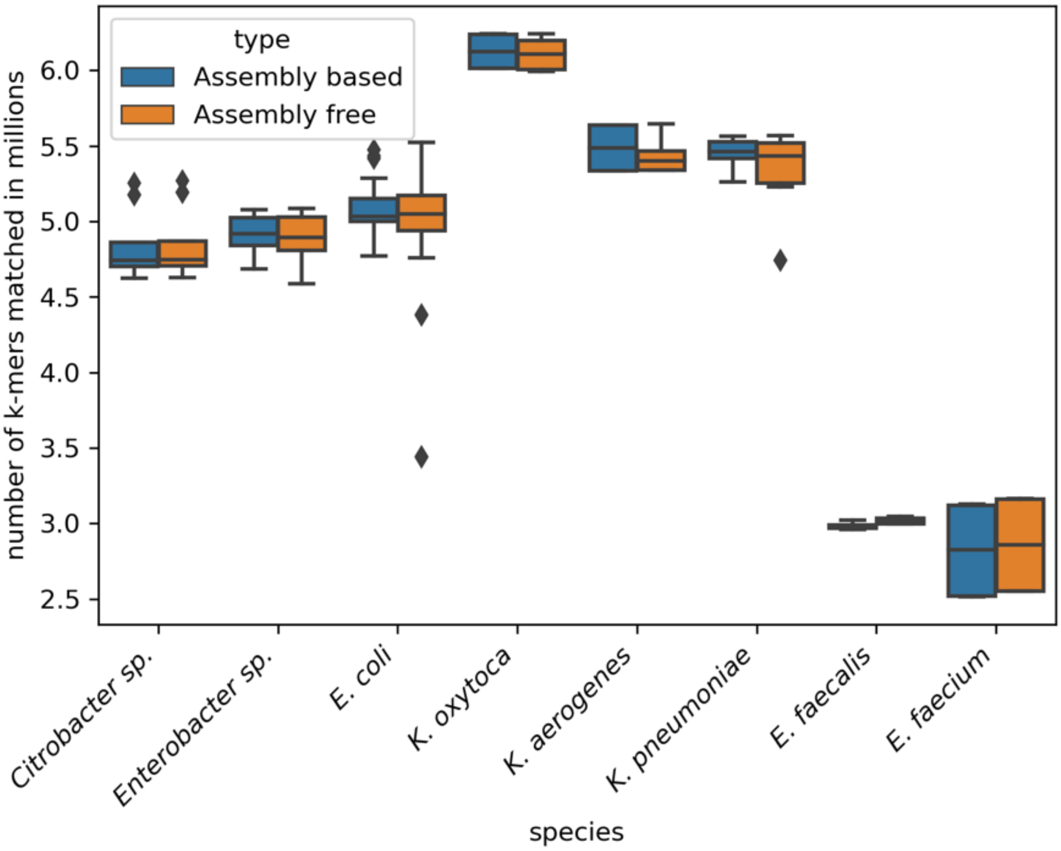

Supplemental figure 3. boxplot of the number of k-mers compared for the assembly free and the assembly based method. Boxes range the interquartile (IQ) range. Whiskers range up to 1.5 times the IQ range. Outliers were shown as single datapoints.
